# Longitudinal associations between youth prosocial behavior and dimensions of psychopathology

**DOI:** 10.1002/jcv2.12282

**Published:** 2024-08-31

**Authors:** Gabrielle E. Reimann, Benjamin B. Lahey, Hee Jung Jeong, E. Leighton Durham, Camille Archer, Carlos Cardenas‐Iniguez, Marc G. Berman, Tyler M. Moore, Brooks Applegate, Antonia N. Kaczkurkin

**Affiliations:** ^1^ Department of Psychology Vanderbilt University Nashville Tennessee USA; ^2^ Departments of Health Studies and Psychiatry and Behavioral Neuroscience University of Chicago Chicago Illinois USA; ^3^ Department of Population and Public Health Sciences Keck School of Medicine of University of Southern California Los Angeles California USA; ^4^ Department of Psychology University of Chicago Chicago Illinois USA; ^5^ Grossman Institute for Neuroscience, Quantitative Biology and Human Behavior University of Chicago Chicago Illinois USA; ^6^ Department of Psychiatry Perelman School of Medicine University of Pennsylvania Philadelphia Pennsylvania USA; ^7^ Department of Educational Leadership, Research and Technology Western Michigan University Kalamazoo Michigan USA

**Keywords:** attention‐deficit/hyperactivity disorder, conduct problems, general psychopathology, internalizing, prosocial behavior

## Abstract

**Background:**

Studies suggest that prosocial behavior, having high empathy and engaging in behaviors intended to benefit others, may predict mental health or vice versa; however, these findings have been mixed. The purpose of the current study was to examine the bidirectional relationships between prosocial behavior and dimensions of psychopathology in children.

**Methods:**

The relationships between prosocial behavior and four dimensions of psychopathology (general psychopathology, internalizing symptoms, conduct problems, and attention‐deficit/hyperactivity disorder symptoms) were examined longitudinally in children 9–12 years of age from the Adolescent Brain Cognitive Development Study (*N* = 9122). We used a random intercept cross‐lagged panel model to distinguish between stable, trait‐like (between‐person) and time‐dependent (within‐person) fluctuations across a 24‐month period.

**Results:**

Between‐person results revealed that prosocial behavior was negatively associated with general psychopathology and conduct problems while being positively associated with internalizing symptoms. Within‐person results demonstrated that, out of four possible directional paths tested, one was significant. This path showed that greater general psychopathology and conduct problems at the first‐year follow‐up predicted fewer prosocial behaviors at the second‐year follow‐up, although the effect size was small. In contrast, prosocial behavior did not predict psychopathology dimensions for any year.

**Conclusions:**

The results of this study suggest that prosocial behaviors have stable associations with psychopathology across preadolescence; however, evidence of a directional association in which psychopathology predicts fewer prosocial behaviors in the future was only modest.

## Graphical abstract

Using a random intercept cross‐lagged panel model, we examined the bidirectional associations between prosocial behavior and youth psychopathology. General psychopathology and conduct problems unidirectionally predicted less future prosocial behavior, suggesting that only some forms of psychopathology are predictive of prosociality.

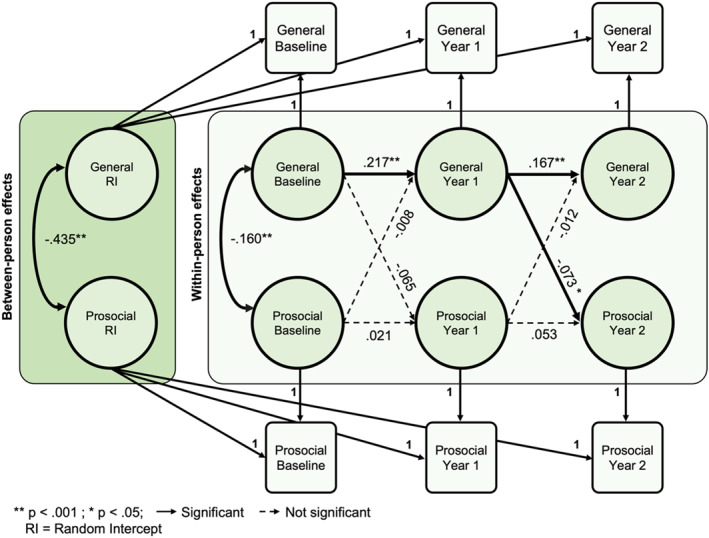

**Key points**

- Prosocial behavior, characterized by high empathy and actions intended to benefit others, is linked to mental health outcomes; however, the nature and direction of this association remain unclear.
- This study employed a longitudinal design with a large sample and utilized advanced statistical modeling to differentiate between stable, trait‐like associations and time‐dependent fluctuations of prosocial behavior and psychopathology dimensions in children.
- Stable prosocial behavior was negatively associated with general psychopathology and conduct problems, and positively associated with internalizing symptoms. Higher general psychopathology and conduct problems predicted fewer prosocial behaviors in the subsequent year.
- These findings suggest the possibility of developing early interventions for enhancing prosocial skills in children with high psychopathology to promote better mental health and social functioning in youth.

## INTRODUCTION

Prosocial behavior refers to the engagement in socioemotional behaviors intended to benefit others, such as empathy, cooperation, helpfulness, and consideration of the feelings of others. These behaviors emerge in infancy and grow in complexity throughout the lifespan. Infant pointing is thought to serve as an early indicator of cooperation via information sharing (Liszkowski, 2005). Toddlers show distress and empathic concern upon witnessing another's sadness (Bandstra et al., 2011). By early childhood, prosocial behaviors are linked to a broad range of areas, from adaptive abilities like social skills to more abstract concepts like morality (Eron & Huesmann, 1984; Fabes et al., 1999; Groeben et al., 2011). These behaviors reflect individual differences in social, cognitive, and affective processes, making them a clinically relevant focus in psychopathology research (Do et al., 2019; Fabes et al., 1999; Knafo & Plomin, 2006; Knafo et al., 2008; Ramot et al., 2020).

Studies show that less prosocial behavior is linked to greater conduct disorder traits (e.g., aggression, callous‐unemotional traits) and attention‐deficit/hyperactivity disorder (ADHD) symptoms (Flouri & Sarmadi, 2016; Huber et al., 2019; Lahey et al., 2018; Perren et al., 2007). Prosocial behavior may have clinical utility as a predictor of long‐term psychopathology as cooperativeness in early childhood is associated with fewer externalizing problems in later childhood and adolescence (Hay & Pawlby, 2003; Padilla‐Walker et al., 2015). Engaging in fewer prosocial behaviors has been linked to later life aggression, behavioral problems, and antisocial personality disorder (Eron & Huesmann, 1984; Lahey et al., 2018). There is also evidence that prosocial behavior has a bidirectional relationship with externalizing psychopathology (Memmott‐Elison & Toseeb, 2022). In contrast, engagement in prosocial behaviors may be related to higher levels of internalizing psychopathology. Increased cooperation and greater concern for others have been associated with more internalizing symptoms, including depression and anxiety (Nantel‐Vivier et al., 2014; Perren et al., 2007). However, other studies report the opposite effect or no significant association between prosocial behaviors and internalizing symptoms (Flouri & Sarmadi, 2016; Hay & Pawlby, 2003; Perren & Alsaker, 2009).

Inconsistent associations between prosocial behaviors and psychopathology may be due to the reliance on diagnostic categories that do not take into account the dimensional and hierarchical nature of mental health symptoms. Hierarchical modeling can be used to identify a common factor of psychopathology (the p factor or general psychopathology factor) representing symptoms common across all disorders, as well as subfactors reflecting specific dimensions, such as internalizing symptoms, conduct problems, and ADHD symptoms (Durham et al., 2021; Kaczkurkin et al., 2019; Lahey et al., 2017). Such an approach can take into account the high comorbidity that exists among mental health disorders (Gnanavel et al., 2019; Kessler et al., 2005). Using this hierarchical approach, we can capture the dimensional nature of psychopathology while also examining the unique associations between specific dimensions of psychopathology and prosocial behavior.

The current study aimed to investigate the longitudinal relationship between psychopathology dimensions and prosocial behavior, incorporating several advances over prior work. First, we utilized a large sample of over 9000 youth from the Adolescent Brain Cognitive Development^SM^ Study (ABCD Study^®^). Second, we used hierarchical modeling to define four dimensions of psychopathology (general psychopathology, internalizing symptoms, conduct problems, and ADHD symptoms). Finally, we employed an advanced statistical technique, random intercept cross‐lagged panel modeling (RI‐CLPM), to distinguish stable, trait‐like (between‐person) associations from time‐dependent (within‐person) changes in psychopathology symptoms and prosocial behaviors over 24 months. We hypothesized a bidirectional effect between conduct problems and prosocial behavior: higher levels of conduct problems would be associated with fewer prosocial behaviors later, and conversely, lower levels of prosocial behavior would be linked to greater future conduct problems. Given the mixed results of prior studies on internalizing psychopathology, examining the relationship between internalizing symptoms and prosocial behavior was exploratory.

## METHODS

### Participants

We used data from the ABCD Study at baseline (9–10 years), the first‐year follow‐up (10–11 years), and the second‐year follow‐up (11–12 years) from release 4.0. The ABCD Study recruited participants from 21 sites across the United States using probability sampling of schools within independent catchment areas while considering factors like age, sex, race/ethnicity, socioeconomic status, and urbanicity. The ABCD Study sites are not perfectly representative of the U.S. population; therefore, post‐stratification weights were applied to make the sample more representative in terms of demographics. After excluding those missing data on the measures of interest, the final sample size for analyses was 9122 participants. The demographic characteristics of the final sample at baseline are summarized in Table 1. Caregiver consent and minor assent were obtained from all participants. The Vanderbilt University institutional review board approved the use of this de‐identified data.

**TABLE 1:** Demographic characteristics of the sample (*N* = 9122).

	*n*	%
Sex		
Male	4756	52.0
Female	4366	48.0
Race‐ethnicity		
Non‐Hispanic White	5016	55.0
African American/Black	1174	12.9
Hispanic	1812	19.9
Asian	186	2.0
Multiracial/other	934	10.2
Income		
<$5000	244	2.7
$5000–$11,999	277	3.0
$12,000–$15,999	191	2.1
$16,000–$24,999	371	4.1
$25,000–$34,999	481	5.3
$35,000–$49,999	698	7.6
$50,000–$74,999	1167	12.8
$75,000–$99,999	1301	14.2
$100,000–$199,999	2699	29.6
≥$200,000	992	10.9
Missing	701	7.7
Parent education		
No degree	389	4.3
High school/GED	977	10.7
Some college	1434	15.7
Associate degree	1182	13.0
Bachelor's degree	2736	30.0
Master's degree	1837	20.1
Professional/Doctoral	567	6.2

	*M*	*SD*
Baseline		
Age (years)	9.90	0.63
Prosocial behavior	10.50	2.44
First‐year follow‐up		
Age (years)	10.9	0.65
Prosocial behavior	10.34	2.49
Second‐year follow‐up		
Age (years)	12.0	0.66
Prosocial behavior	10.31	2.56

*Note*: The “Multiracial/other” category includes those who were identified by their caregiver as American Indian/Native American, Alaska Native, Native Hawaiian, Guamanian, Samoan, Other Pacific Islander, Asian Indian, Chinese, Filipino, Japanese, Korean, Vietnamese, Other Asian, or Other Race.

Abbreviations: GED, general education development; M, mean; SD, standard deviation.

### Measures

#### Psychological symptoms

The Child Behavior Checklist (CBCL) is a caregiver rating scale consisting of 119 items describing child behaviors and emotions (Achenbach, 2009). We used the model described in Moore et al. (2020) to define the hierarchical dimensions of psychopathology. This model identified four orthogonal psychopathology dimensions based on the CBCL data at baseline, first‐year follow up, and second‐year follow up, as detailed in prior work (Moore et al., 2020). Internalizing, conduct problems, and ADHD dimensions were identified through exploratory factor analysis and confirmed with a bifactor model, which showed adequate construct reliability and estimated replicability (Moore et al., 2020). The items positively loading onto general psychopathology (66 items), internalizing symptoms (19 items), conduct problems (32 items), and ADHD symptoms (15 items) are detailed elsewhere (Moore et al., 2020).

#### Prosocial behavior

Prosocial behavior was operationalized by a sum score of the three items from the Prosociality Scale of the Strength and Difficulties Questionnaire (SDQ) Caregiver Report: (1) considerate of others' feelings, (2) helpful if someone is hurt, and (3) offers help to others (Goodman, 1997). These questions are rated by caregivers on a scale of 0 = *not true*, 1 = *somewhat true*, or 2 = *certainly true*.

### Statistical analyses

To examine the longitudinal associations between dimensions of psychopathology and prosocial behavior, we applied a RI‐CLPM using data from three measurement waves. This approach allows us to separate the observed variance in prosocial behavior and each psychopathology dimension to differentiate between stable traits (between‐person effects) and temporal fluctuations (within‐person effects) over a 24 month period (Mulder & Hamaker, 2021). Between‐person analyses assess consistent deviations from the grand means, represented by two random intercepts that quantify the associations between trait‐like aspects of psychopathology and prosocial behavior over time, with factor loadings fixed to 1. Each random intercept represents a latent variable created from prosocial behavior and psychopathology dimensions across three waves. Within‐person components capture deviations between an individual's observed measurements and their expected scores at each time point (*t*). Cross‐lagged within‐person analyses examine associations from one time point (*t*) to the next (*t* + 1). For example, we examined associations from baseline psychopathology to first‐year prosocial behavior and vice versa. We tested whether deviations from the expected behavior at time *t* + 1 (e.g., expected prosocial behavior at the first‐year follow‐up) were predicted by separate behaviors at time *t* (e.g., baseline psychopathology). The associations between each dimension of psychopathology (general psychopathology, internalizing symptoms, conduct problems, and ADHD symptoms) and prosocial behavior were examined in separate models. The RI‐CLPM was conducted using Mplus version 8.3, applying the mean‐ and variance‐adjusted weighted least squares (WSLMV) estimator and pairwise deletion for missing data. Model fit was assessed using the following criteria: comparative fit index (CFI) ≥ 0.9, root mean square error of approximation (RMSEA) ≤ 0.06, and standardized root mean square residual (SRMR) ≤ 0.08 (Jackson et al., 2009; McDonald & Ho, 2002).

Analyses accounted for the stratification of the sample in data collection sites using the “strat” command in Mplus. This approach is recommended for the analysis of complex survey data and has been used previously in publishing with ABCD Study data (Durham et al., 2021; Jeong et al., 2023; Muthén, L. K., & Muthén, 2017; Reimann et al., 2023, 2024). Analyses also employed post‐stratification weights to make the sample more representative of the general population and accounted for clustering of siblings/multiple births (twins/triplets) within families. In addition, we divided the sample into males and females to examine sex differences. We controlled for the false discovery rate (*q* < 0.05) using the stats package in R version 4.3.1 (http://www.r‐project.org/). The ABCD Study is a publicly available dataset that can be accessed through the National Institute of Mental Health Data Archive (https://nda.nih.gov/abcd; DOI: 10.15154/1523041). Code and analytic procedures can be found at https://github.com/VU‐BRAINS‐lab/Reimann_Prosocial_RICLPM.

## RESULTS

### Prosocial scale reliability

Prosocial items at each time point showed sufficient endorsement across responses. Using the alpha coefficient and omega coefficient at each time point, the Prosociality Scale showed good internal consistency at baseline (*∝* = 0.80, *ω* = 0.80), first‐year follow‐up (*∝* = 0.80, *ω* = 0.81), and second‐year follow‐up (*∝* = 0.81, *ω* = 0.81) (Cronbach & Meehl, 1955; Dunn et al., 2014). Additionally, the range of variance for the prosocial items was similar across time points (baseline: 0.20–0.28, first‐year follow‐up: 0.19–0.27, second‐year follow‐up: 0.19–0.29), demonstrating consistent response patterns over time, which supports the reliability of the scale.

### General psychopathology at year 1 predicts prosocial behaviors at year 2

**FIGURE 1:**
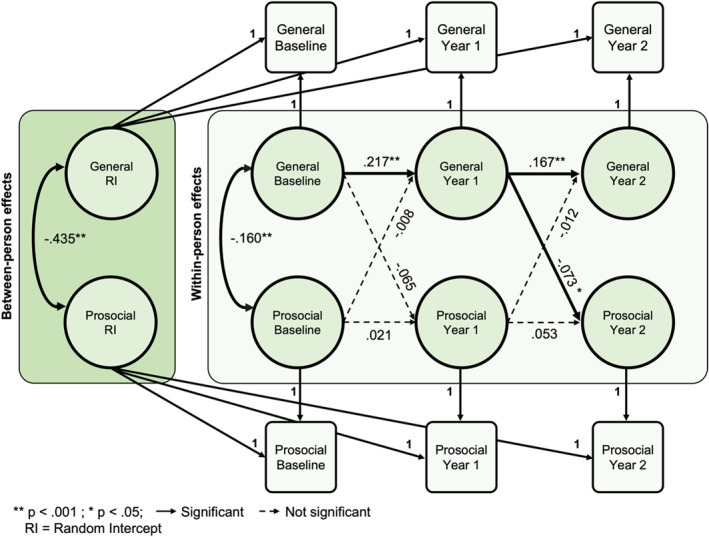
Random intercept cross‐lagged panel model of general psychopathology and prosocial behavior.

The RI‐CLPM for general psychopathology and prosocial behavior showed satisfactory fit indices (CFI > 0.99, RMSEA = 0.04, SRMR = 0.01). As expected, general psychopathology was positively associated with subsequent general psychopathology symptoms from baseline to year one (*β* = .217, *p* < .001, *R* ^*2*^ = 0.05) and from year one to year two (*β* = .167, *p* < .001, *R* ^*2*^ = 0.03; Figure 1). Prosocial behavior was not associated with prosocial behavior at subsequent time points, despite strong internal consistency among scale items. Between‐person effects showed that individuals with greater general psychopathology symptoms engaged in fewer prosocial behaviors consistently across time (*β* = −.435, *p* < .001). Cross‐lagged effects showed that the path from general psychopathology symptoms at baseline to prosocial behavior at year one was not significant. However, there was a small negative association between general psychopathology at year one and prosocial behavior at year two (*β* = −.073, *p* = .02, *R* ^*2*^ = 0.01). This indicates that greater psychopathology symptoms at the first‐year follow‐up were associated with lower prosocial behaviors in the subsequent year. There were no significant cross‐lagged paths from prosocial behavior to general psychopathology.

When analyzing psychopathology and prosocial behavior by sex, cross‐lagged results remained the same among males, showing a unidirectional association between general psychopathology symptoms at year one and prosocial behavior at year two (*β* = −.098, *p* = .028, *R* ^*2*^ = 0.01; Figure S1). However, cross‐lagged paths from general psychopathology to prosocial behavior were not significant across any time point among females (Figure S2).

### Conduct problems at year 1 predict prosocial behaviors at year 2

**FIGURE 2:**
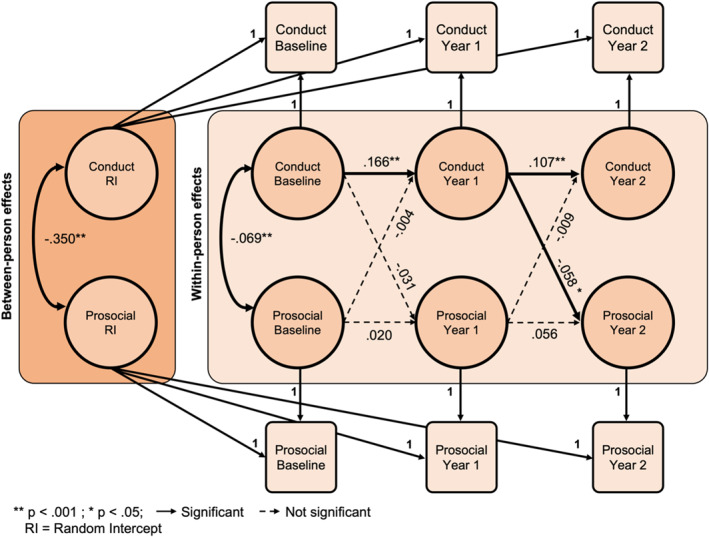
Random intercept cross‐lagged panel model of conduct problems and prosocial behavior.

The fit indices for the RI‐CLPM between conduct problems and prosocial behavior were satisfactory (CFI > 0.99, RMSEA = 0.03, SRMR < 0.01). Conduct problems predicted future conduct problems from baseline to year one (*β* = .166, *p* < .001, *R* ^*2*^ = 0.03) and from year one to year two (*β* = .107, *p* < .001, *R* ^*2*^ = 0.01; Figure 2). Between‐person effects showed that those with greater conduct problems endorsed fewer prosocial behaviors consistently over time (*β* = −.350, *p* < .001, *R* ^*2*^ = 0.12). As with general psychopathology, a single cross‐lagged effect was apparent with greater conduct problems at year one predicting lower prosocial behavior at year two (*β* = −.058, *p* = .028, *R* ^*2*^ = 0.003), but no significant effect from baseline to year one. There were no significant cross‐lagged paths from prosocial behavior to conduct problems.

Cross‐lagged results were consistent among males, showing a unidirectional association where conduct problems at year one predicted prosocial behavior at year two (*β* = −.080, *p* = .036, *R* ^*2*^ = 0.01; Figure S3). However, the cross‐lagged results did not show significant associations between conduct problems and prosocial behavior at any time point for females (Figure S4).

### Internalizing symptoms do not predict subsequent prosocial behaviors

**FIGURE 3:**
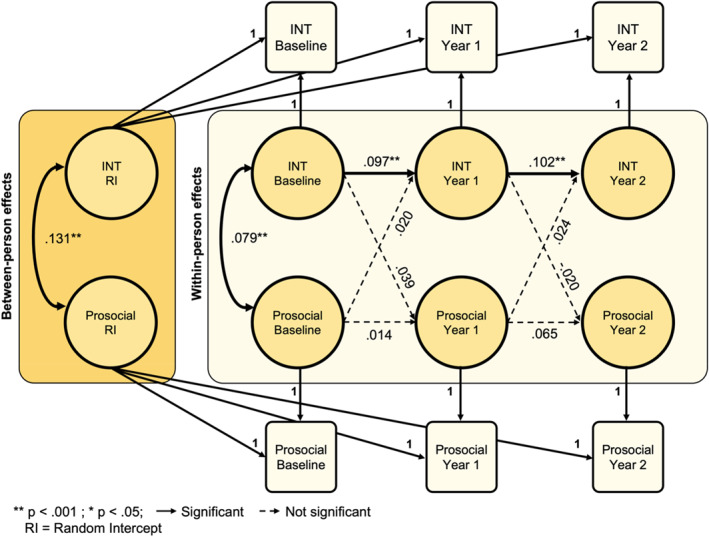
Random intercept cross‐lagged panel model of internalizing symptoms and prosocial behavior. INT, internalizing.

The fit indices for the RI‐CLPM between internalizing symptoms and prosocial behavior were satisfactory (CFI > 0.99, RMSEA < 0.01, SRMR < 0.01). Internalizing symptoms predicted future internalizing symptoms from baseline to year one (*β* = .097, *p* < .001, *R* ^*2*^ = 0.01) and from year one to year two (*β* = .102, *p* < .001, *R* ^*2*^ = 0.01; Figure 3). Between‐person effects showed that individuals with greater internalizing symptoms endorsed greater prosocial behavior consistently across time (*β* = .131, *p* < .001, *R* ^*2*^ = 0.02). No significant cross‐lagged effects emerged between internalizing symptom endorsement and prosocial behavior at subsequent time points, despite satisfactory fit indices. Analyses by sex showed that the results remained similar for males and females (Figures S5 and S6).

### ADHD symptoms show no relationship with prosocial behavior

**FIGURE 4:**
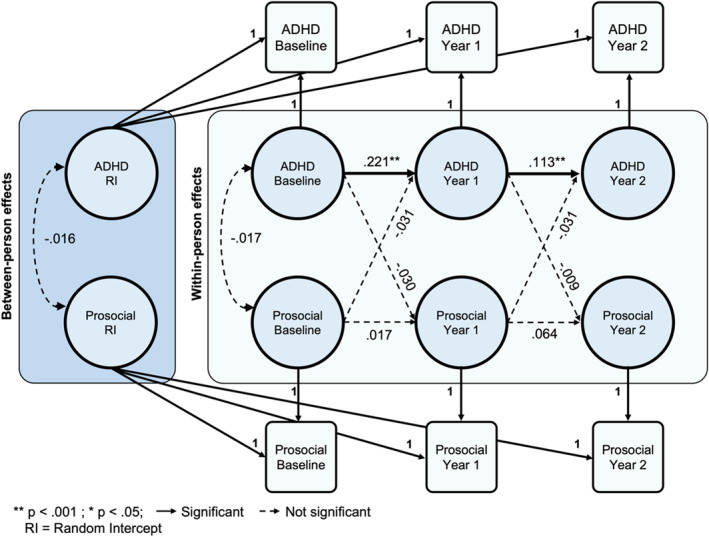
Random intercept cross‐lagged panel model of ADHD symptoms and prosocial behavior. ADHD, attention‐deficit/hyperactivity disorder.

Fit indices were adequate for the RI‐CLPM between ADHD symptoms and prosocial behavior (CFI > 0.99, RMSEA < 0.02, SRMR < 0.01). ADHD symptoms at each time point predicted subsequent ADHD symptoms, from baseline to year one (*β* = .221, *p* < .001, *R* ^*2*^ = 0.05) and from year one to year two (*β* = .113, *p* < .001, *R* ^*2*^ = 0.01; Figure 4). There was no between‐person association between prosocial behavior and ADHD symptom endorsement. No significant cross‐lagged effects emerged between ADHD and prosocial behavior at subsequent time points. Analyses by sex indicated that the results were consistent across both males and females (Figures S7 and S8).

## DISCUSSION

The current study examined the bidirectional relationship between psychopathology and prosocial behavior in children, differentiating between stable (between‐person) features and time‐dependent changes (within‐person) across multiple time points. Greater general psychopathology symptoms and conduct problems were associated with less prosocial engagement, consistent with prior work (Eron & Huesmann, 1984; Flouri & Sarmadi, 2016; Frick et al., 2005; Hay & Pawlby, 2003; Huber et al., 2019; Lahey et al., 2018; Perren et al., 2007; Robertson et al., 2020). In contrast, greater internalizing symptoms were associated with greater prosocial engagement. These between‐person effects did not differ by sex. We also found that greater general psychopathology symptoms and conduct problems at the first‐year follow‐up predicted lower prosocial engagement at the second‐year follow‐up. These effects were significant for males but not females. No significant paths were found from prosocial behavior to psychopathology for any of the psychopathology dimensions. The results of this study show that while stable relationships exist between psychopathology and prosocial behavior, the direction of these effects depends on the type of symptoms experienced. Furthermore, this study suggests that early signs of general psychopathology symptoms and conduct problems may lead to a decline in prosocial behavior over time, emphasizing the potential long‐term impact of mental health symptoms on children's socioemotional development.

A key finding of the current study was that general psychopathology symptoms and conduct problems predicted a reduction in prosocial behavior from the first‐to the second‐year follow‐up, suggesting that the association between psychopathology and future prosocial behavior may become more pronounced as symptoms increase with age. This association was primarily observed in males in the current study, which may be expected given that conduct problems are more prevalent among males than females. The emergence of conduct problem symptoms may hinder the development of prosocial tendencies over time, potentially leading to a negative cascade effect. For example, engaging in aggressive behavior and rule violations may impair a child's ability to process distress signals and develop empathy, ultimately reducing prosocial behavior (Decety et al., 2016). The results of the current study are consistent with prior research demonstrating that behavioral problems are a stronger predictor of subsequent prosocial behavior than vice versa. Memmott‐Elison and Toseeb (2022) found that conduct problems predicted prosocial behavior 2 years later, whereas prosocial behavior did not significantly predict conduct problems until mid‐childhood, and even then, the association was modest. The absence of a predictive association between low prosocial behavior and future conduct problems does not imply that interventions that increase prosocial behavior could not still play a valuable role in preventing the development of externalizing problems in youth. Future work is needed that examines the impact of prosocial behavioral modification on subsequent psychopathology outcomes.

Prior work shows mixed findings regarding the association between prosocial behavior and internalizing psychopathology (Dalsgaard et al., 2020; Flouri & Sarmadi, 2016; Hay & Pawlby, 2003; Perren & Alsaker, 2009; Perren et al., 2007). Our between‐person results demonstrated that greater internalizing symptoms are associated with more engagement in prosocial behavior, although internalizing symptoms did not predict changes in future prosocial behavior. This positive association contrasts with a recent meta‐analysis reporting that greater internalizing symptoms were associated with fewer prosocial behaviors (Memmott‐Elison et al., 2020). This divergence in findings may be attributed to our hierarchical modeling approach. In a bifactor model, the general psychopathology factor captures the shared variance across all psychopathology domains, leaving the residual variance in each subfactor (i.e., ADHD, conduct problems, and internalizing symptoms). Thus, internalizing symptoms in our model are not comparable to internalizing symptoms found in prior studies. The internalizing symptoms in this study reflect what is unique to internalizing disorders after accounting for the common symptoms captured by the general factor. Our results suggest that the prior negative association found between internalizing symptoms and prosocial behavior may be attributed to the general factor, while specific internalizing symptoms show a positive relationship with prosocial behavior. This is consistent with studies demonstrating that prosocial behaviors including greater empathy, engagement with others, and friendliness have been associated with internalizing psychopathology (Bohlin et al., 2000; de Sousa et al., 2021). Our hierarchical modeling approach helps to disentangle the mixed findings in the literature on internalizing symptoms and prosocial behavior by showing both a general negative association (via a general psychopathology factor) and a specific positive association (via a specific internalizing factor).

The current study found no significant associations at the between‐ or within‐person levels for ADHD symptoms. Prior studies have examined prosocial engagement in ADHD in conjunction with conduct problems under the umbrella term “externalizing symptoms.” However, research on ADHD symptoms reveals unique characteristics separate from conduct problems, such as socialization difficulties driven by inattention and preoccupation rather than disregard for the rights of others (Blachman & Hinshaw, 2002; Durham et al., 2021; Hay et al., 2010; Hoza, 2007; Hoza et al., 2000; McQuade & Hoza, 2008; Mikami & Normand, 2015; Moore et al., 2020; Reimann et al., 2023). As such, it is important to evaluate associations between prosocial behaviors and ADHD symptoms specifically. In examining ADHD symptoms separately from conduct problems and general psychopathology, the current study did not find consistent trait or temporal associations with prosocial behaviors. This does not imply that ADHD symptoms are unrelated to prosocial behavior; rather, any potential relationship may be non‐specific and instead reflected in the broader association between general psychopathology and prosocial behavior. These results also underscore an important distinction between conduct problems and ADHD symptoms, suggesting that studies which combine these symptom classes might miss critical differences.

Finally, it is important to note that prosocial behavior showed stable associations with several psychopathology factors but was not predictive of future prosocial behavior in the current study. Additionally, we did not find evidence for bidirectional associations between prosocial behavior and psychopathology. This lack of bidirectional effects contrasts with RI‐CLPM analyses from Memmott‐Elison and Toseeb (2022), who observed that higher levels of psychopathology (peer issues, conduct problems, and hyperactivity/inattention) were associated with fewer future prosocial behaviors, and vice versa, in 16,984 UK children and adolescents (ages 3–14). The bidirectional effects observed in Memmott‐Elison and Toseeb (2022) may be attributed to the longer intervals between assessments (2–4 years) compared to the annual assessments in the current study. It is also important to consider that prosocial behavior involves a complex interplay between biological predisposition and environmental influences. Future work should explore whether environmental attributes moderate these bidirectional associations or impact the stability of prosocial behaviors over time. Additionally, future research could investigate whether increasing prosocial behaviors can mitigate or prevent the development of psychopathology. The context‐dependent nature of prosocial behavior, influenced by both biological and environmental factors, suggests that further research is needed to explore its directional effects and determine how these dynamics can be leveraged to enhance prosocial behavior and positively impact psychopathology outcomes.

Several limitations should be noted. First, our study found small but statistically significant effects in a large sample. Consistent with prior RI‐CLPM work, our results showed greater between‐person effects, while within‐person effects were smaller (Narmandakh et al., 2020). While a RI‐CLPM approach produces robust model fit, a traditional CLPM approach may produce better consistency among cross‐lagged effects (Orth et al., 2020). Second, there are limited prosocial items in the ABCD Study, potentially affecting the lack of association between prosocial behavior at different time points. However, the use of this 3‐item scale is consistent with prior research and has demonstrated specificity with psychopathology (Brislin et al., 2021; Conley et al., 2022). For example, Brislin et al. (2021) found significant differences in prosocial behavior between youth with and without conduct problem symptoms using these items. Despite the widespread use of this scale, there is still a need for a formal definition of prosocial behavior, as this is a relatively new concept in our diagnostic criteria (Narmandakh et al., 2020). Third, while the orthogonality of subfactors in a bifactor model is valuable for isolating unique variance, it can complicate interpretation. Alternative models with clear theoretical justifications may be worth considering (Burke & Johnston, 2020; Burns et al., 2020). Fourth, while the ABCD Study offers a large, well‐defined sample, further research is needed to replicate these findings in other samples. Although the ABCD Study is fairly representative in terms of income when compared to U.S. percentages, there is a slight overrepresentation of individuals in the $100,000–199,999 income bracket. Given prior work suggesting greater economic inequality may be related to lower prosocial behavior, our findings should be interpreted with this in mind (Yang & Konrath, 2023). Fifth, and lastly, we employed a caregiver‐report measure of psychopathology symptoms (CBCL), which is an accurate informant measure for externalizing symptoms but may be less effective for assessing internalizing symptoms that are less outwardly visible to caregivers. The prosocial behavior assessment was also based on caregiver‐report rather than self‐report. Future work would benefit from including self‐report measures of psychopathology symptoms and prosocial behaviors for cross‐informant analyses.

## CONCLUSION

Our findings revealed stable associations between general psychopathology, internalizing symptoms, and conduct problems in relation to prosocial behavior in a large sample of preadolescents. We also identified time‐varying associations between prosocial behavior and both general psychopathology and conduct problems as children age, indicating that broad and conduct‐specific psychopathology symptoms may lead to a diminished regard for others over time. Contrary to previous research, our results did not provide evidence of a bidirectional relationship between prosocial behavior and psychopathology. Further studies are needed to clarify these discrepancies and deepen our understanding of the complex, longitudinal associations between psychopathology and prosocial behavior in youth.

## AUTHOR CONTRIBUTIONS

**Gabrielle E. Reimann**: Data curation; formal analysis; methodology; visualization; writing—original draft; writing—review & editing. **Benjamin B. Lahey**: Conceptualization; resources; writing—review & editing. **Hee Jung Jeong**: Writing—review & editing. **E. Leighton Durham**: Writing—review & editing. **Camille Archer**: Writing—review & editing. **Carlos Cardenas‐Iniguez**: Writing—review & editing. **Marc G. Berman**: Writing—review & editing. **Tyler M. Moore**: Formal analysis; methodology; writing—review & editing. **Brooks Applegate**: Data curation; writing—review & editing. **Antonia N. Kaczkurkin**: Conceptualization; funding acquisition; investigation; project administration; resources; supervision; writing—review & editing.

## CONFLICT OF INTEREST STATEMENT

The authors have declared no competing or potential conflicts of interest.

## ETHICAL CONSIDERATIONS

The use of this deidentified dataset was approved by the Vanderbilt University Institutional Review Board. Prior to conducting the research, the ABCD Study obtained written consent from legal guardians and written assent from the child.

## Supporting information

Supporting Information S1

## Data Availability

Data used in the preparation of this article were obtained from the Adolescent Brain Cognitive Development^SM^ (ABCD) Study (https://abcdstudy.org), held in the NIMH Data Archive (NDA). This is a multisite, longitudinal study designed to recruit more than 10,000 children age 9–10 and follow them over 10 years into early adulthood. The ABCD Study® is supported by the National Institutes of Health and additional federal partners under award numbers U01DA041048, U01DA050989, U01DA051016, U01DA041022, U01DA051018, U01DA051037, U01DA050987, U01DA041174, U01DA041106, U01DA041117, U01DA041028, U01DA041134, U01DA050988, U01DA051039, U01DA041156, U01DA041025, U01DA041120, U01DA051038, U01DA041148, U01DA041093, U01DA041089, U24DA041123, U24DA041147. A full list of supporters is available at https://abcdstudy.org/federal‐partners.html. A listing of participating sites and a complete listing of the study investigators can be found at https://abcdstudy.org/consortium_members/. ABCD consortium investigators designed and implemented the study and/or provided data but did not necessarily participate in the analysis or writing of this report. This manuscript reflects the views of the authors and may not reflect the opinions or views of the NIH, NSF, or ABCD consortium investigators. The ABCD data repository grows and changes over time. The ABCD data used in this report came from RRID: SCR_015769, DOI 10.15154/1523041 (data release 4.0) and NDA study DOI: 10.15154/1528133. DOIs can be found at https://nda.nih.gov/abcd/study‐information.
