# Supplementary material for: Longitudinal associations between youth prosocial behavior and dimensions of psychopathology
Source: JCPP Adv. 2024 Aug 31;5(2):e12282. doi: 10.1002/jcv2.12282 (PMC12159313; doi:10.1002/jcv2.12282)
Supplement: Supplementary file 1 — Supporting Information S1 [file JCV2-5-e12282-s001.docx]

**Supplementary Material for “Longitudinal Associations between Youth Prosocial Behavior and Dimensions of Psychopathology”**

**Table of Contents**

*S1. General psychopathology and prosocial behavior associations among males........................ 2*

*S2. General psychopathology and prosocial behavior associations among females..................... 3*

*S3. Conduct problems and prosocial behavior associations among males........... 4*

*S4. Conduct problems and prosocial behavior associations among females........ 5*

*S5. Internalizing symptoms and prosocial behavior associations among males................ 6*

*S6. Internalizing symptoms and prosocial behavior associations among females.............. 7*

*S7. ADHD symptoms and prosocial behavior associations among males.......................... 8*

*S8. ADHD symptoms and prosocial behavior associations among females....................... 9*


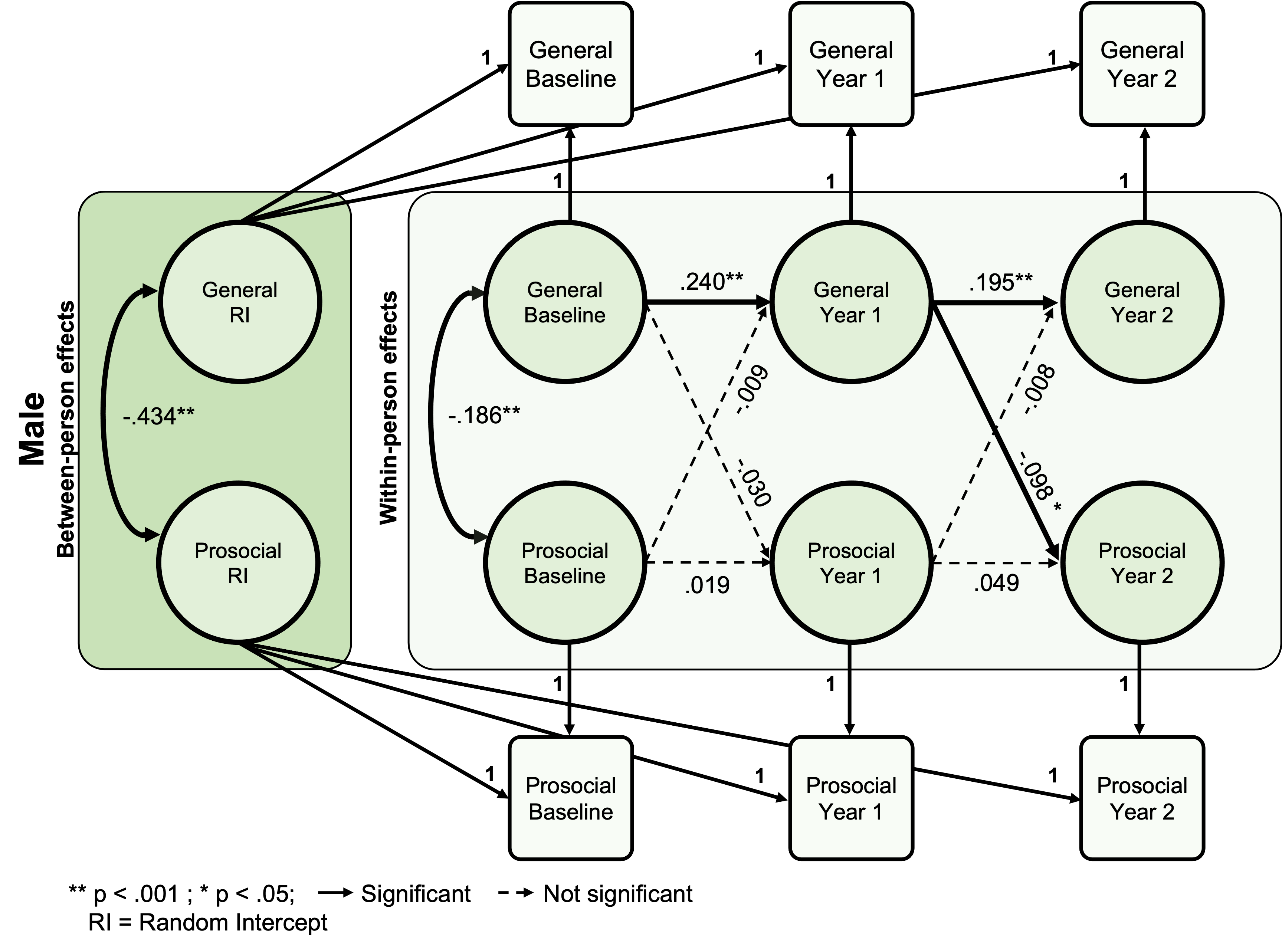


**Figure S1. General psychopathology and prosocial behavior associations among males.** Associations between general psychopathology and prosocial behavior among males across three time points via a random intercept cross-lagged panel model (RI-CLPM). Results in males remain similar to the main analyses.


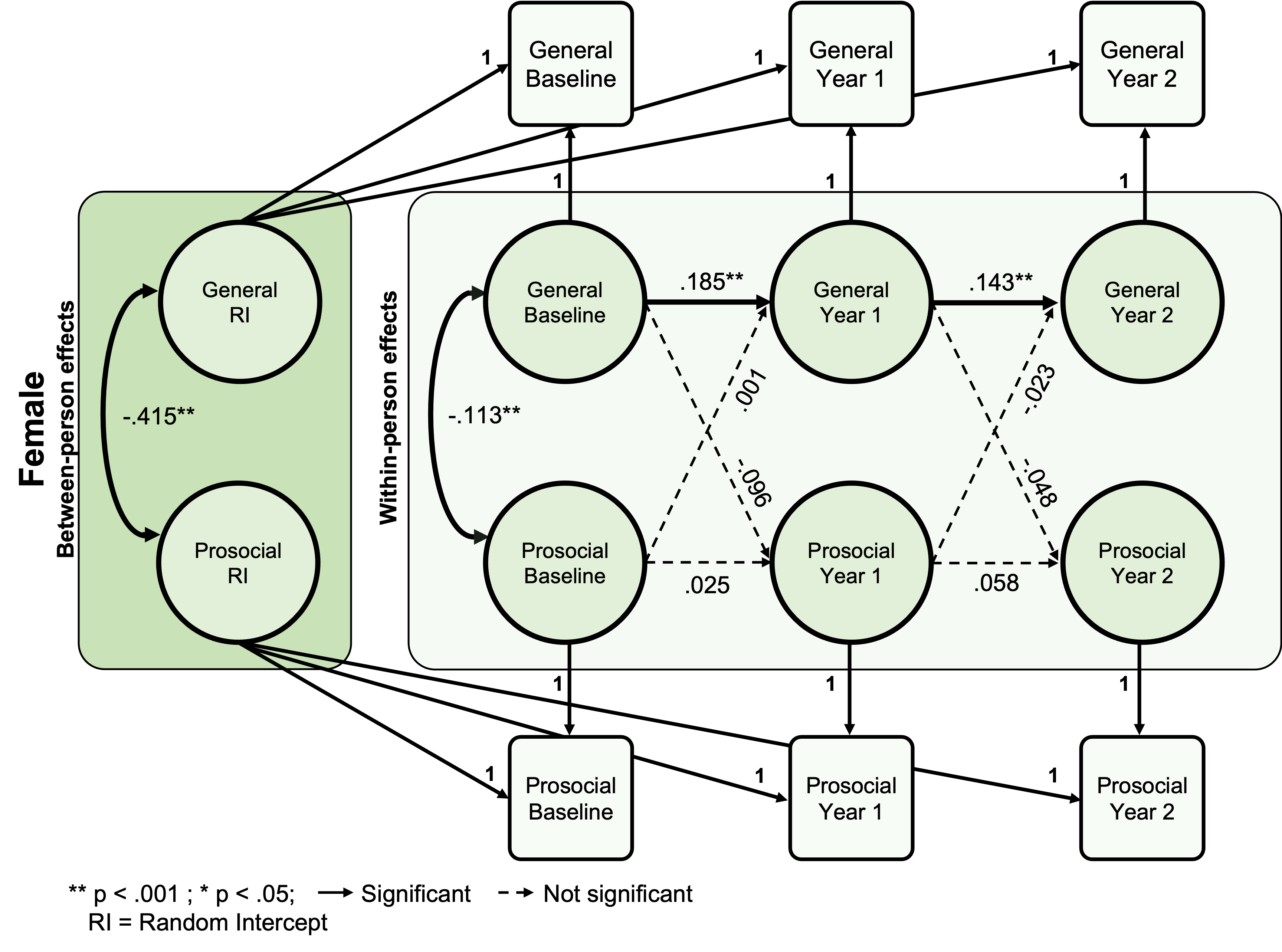


**Figure S2. General psychopathology and prosocial behavior associations among females.** Associations between general psychopathology and prosocial behavior among females across three time points via a random intercept cross-lagged panel model (RI-CLPM). No significant cross-lagged paths were apparent for females.


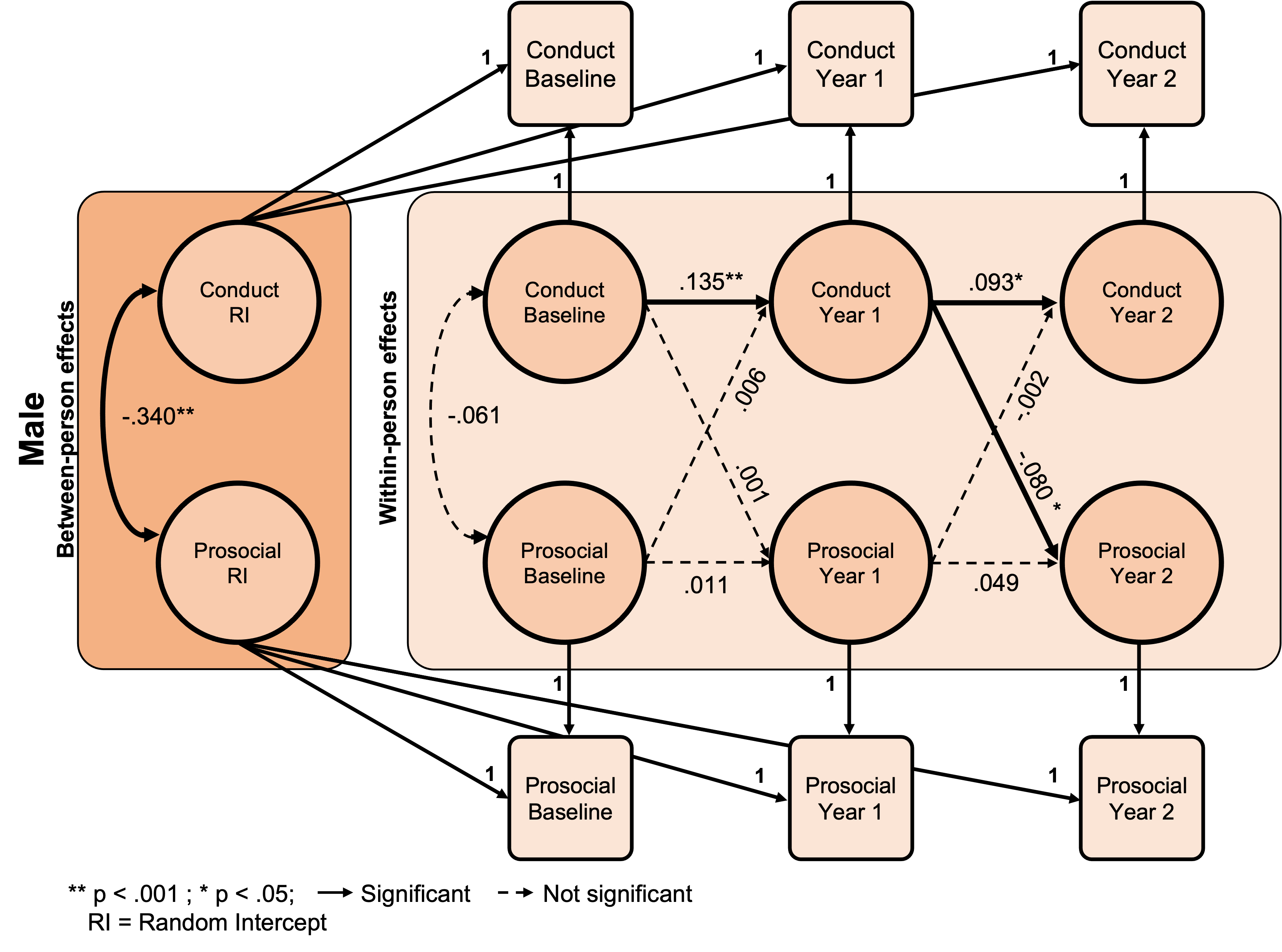


**Figure S3. Conduct problems and prosocial behavior associations among males.** Associations between conduct problems and prosocial behavior among males across three time points via a random intercept cross-lagged panel model (RI-CLPM). Results in males are similar to the main analyses.


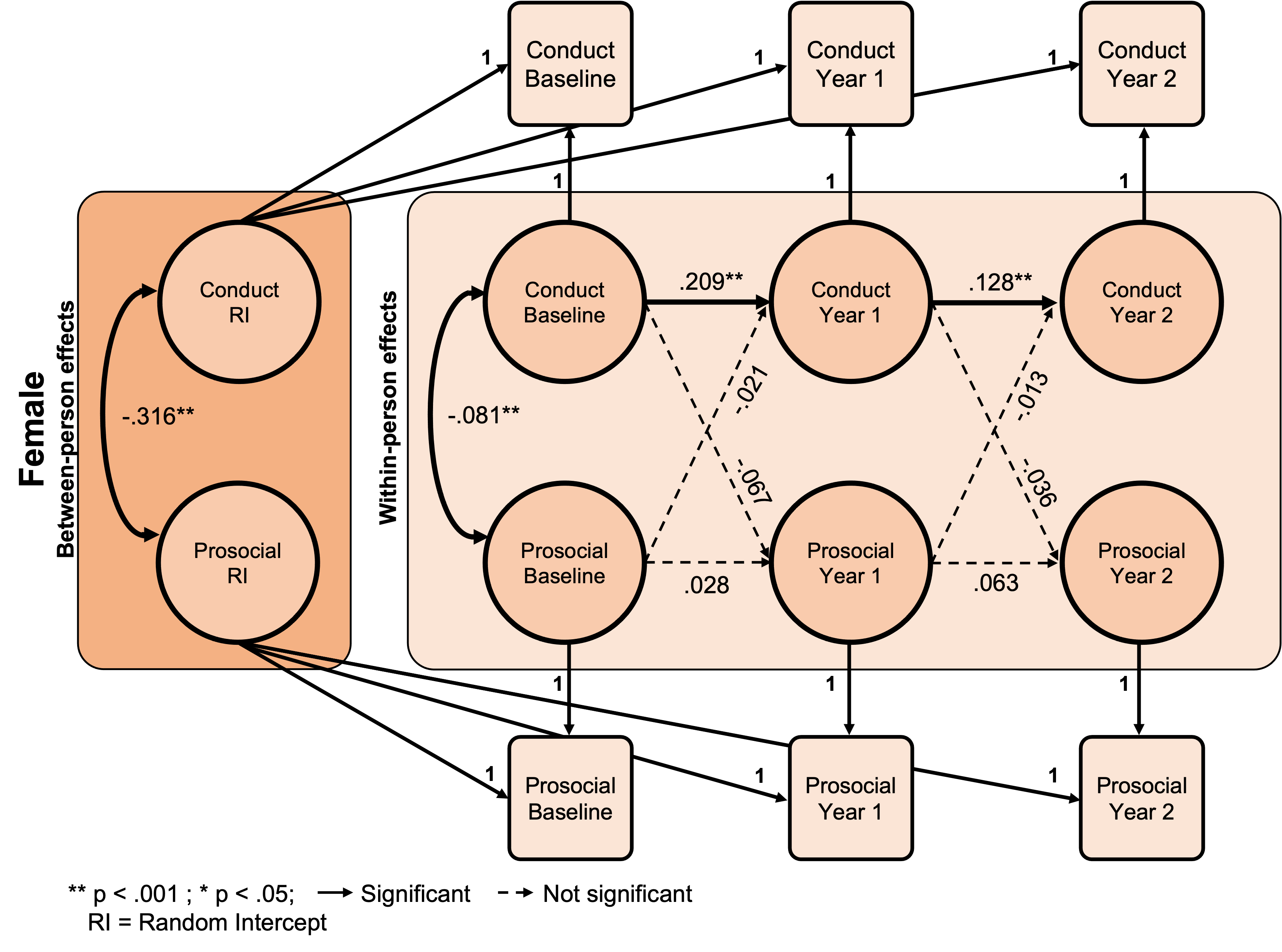


**Figure S4. Conduct problems and prosocial behavior associations among females.** Associations between conduct problems and prosocial behavior among females across three time points via a random intercept cross-lagged panel model (RI-CLPM). No cross-lagged paths were significant for females.


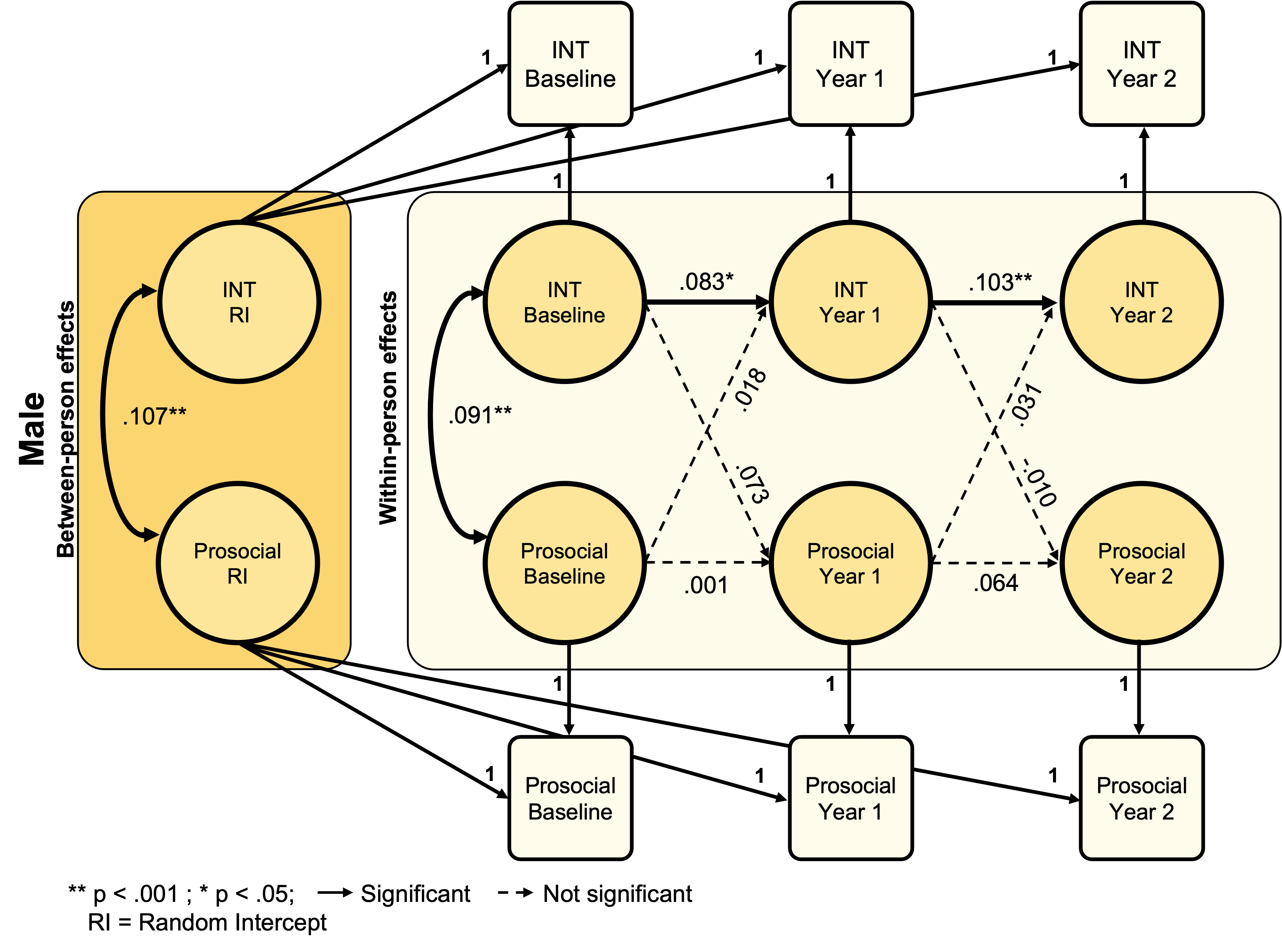


**Figure S5. Internalizing symptoms and prosocial behavior associations among males.** Associations between internalizing symptoms and prosocial behavior among males across three time points via a random intercept cross-lagged panel model (RI-CLPM). Results are similar to the main analyses.


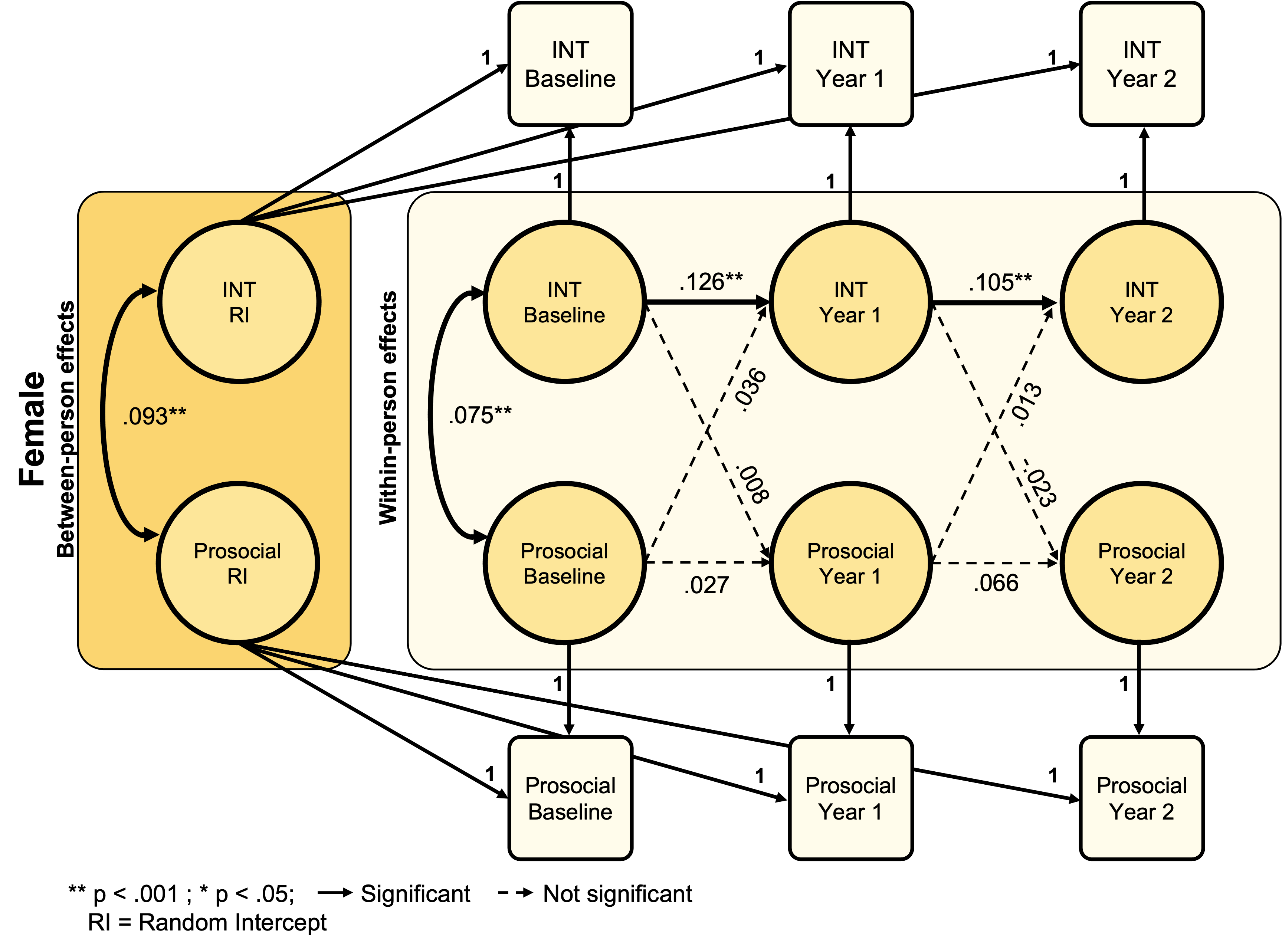


**Figure S6. Internalizing symptoms and prosocial behavior associations among females.** Associations between internalizing symptoms and prosocial behavior among females across three time points via a random intercept cross-lagged panel model (RI-CLPM). Results are similar to the main analyses.


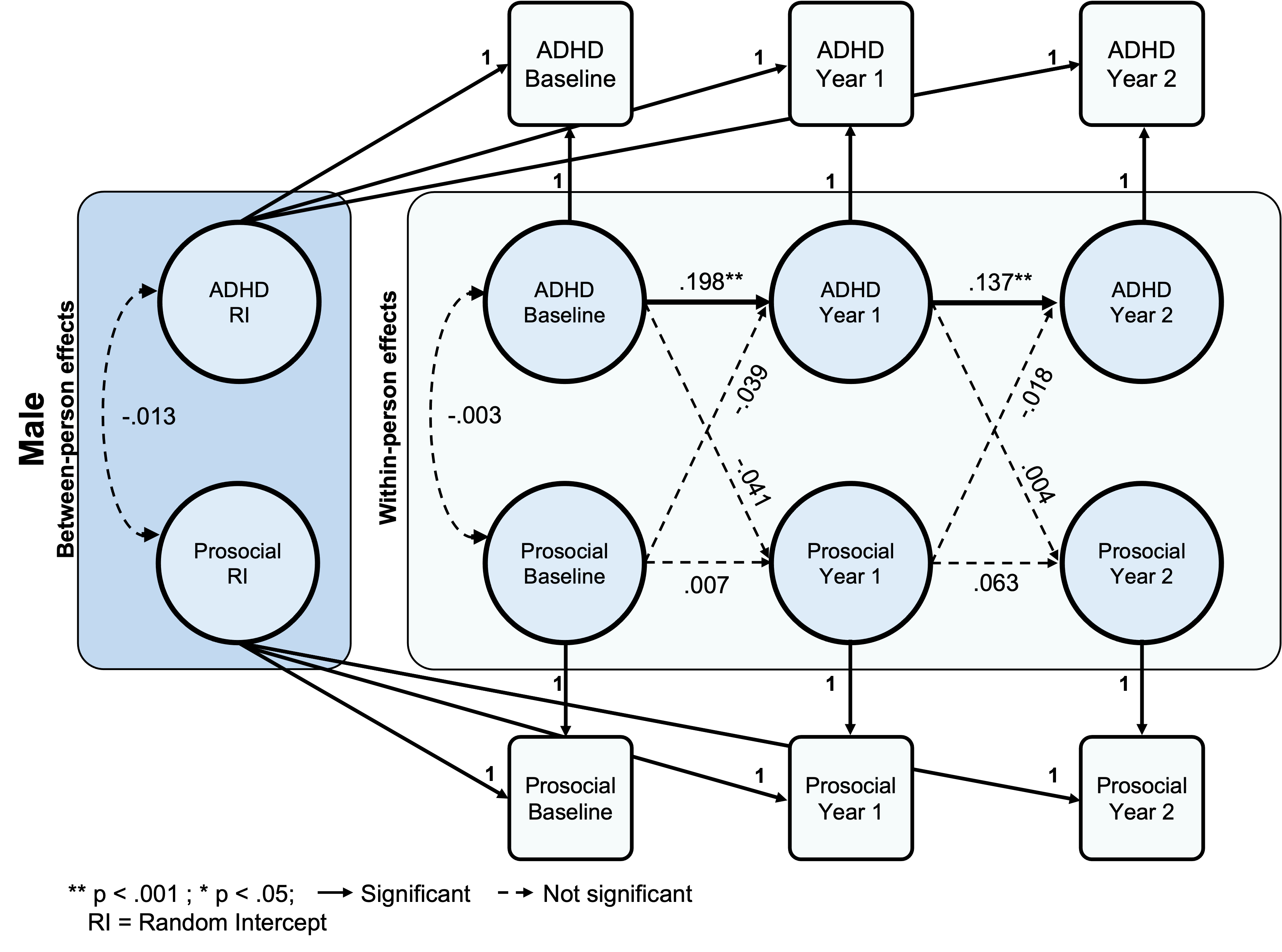


**Figure S7. ADHD symptoms and prosocial behavior associations among males.** Associations between attention-deficit/hyperactivity disorder (ADHD) symptoms and prosocial behavior among males across three time points via a random intercept cross-lagged panel model (RI-CLPM). Results for males are similar to the main analyses.


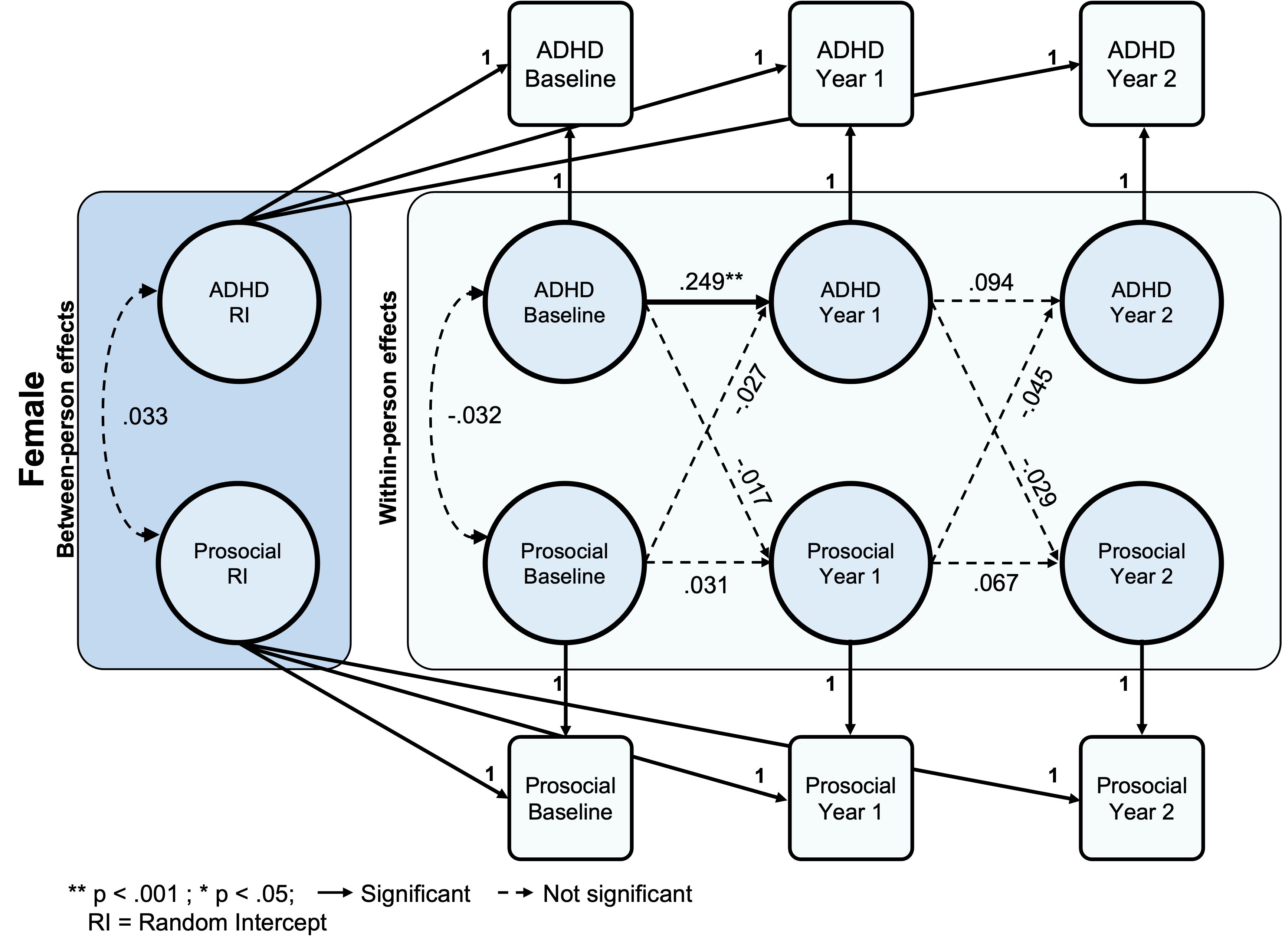


**Figure S8. ADHD symptoms and prosocial behavior associations among females.** Associations between attention-deficit/hyperactivity disorder (ADHD) symptoms and prosocial behavior among females across three time points via a random intercept cross-lagged panel model (RI-CLPM). Results for females show one less significant path between ADHD at year 1 and ADHD at year 2 compared to the main analyses.
